# Composition, Seasonal Dynamics and Metabolic Potential of the Rhizosphere Microbiome Associated with Wild White Poplar

**DOI:** 10.3390/biotech13040052

**Published:** 2024-12-01

**Authors:** Mikhail I. Popchenko, Dmitry S. Karpov, Natalya S. Gladysh, Maxim A. Kovalev, Vsevolod V. Volodin, George S. Krasnov, Alina S. Bogdanova, Nadezhda L. Bolsheva, Maria S. Fedorova, Anna V. Kudryavtseva

**Affiliations:** 1 Engelhardt Institute of Molecular Biology, Russian Academy of Sciences, 32 Vavilova, 119991 Moscow, Russia; popchenko_m@mail.ru (M.I.P.); natalyagladish@gmail.com (N.S.G.); kovalev_maksim_2002@mail.ru (M.A.K.); vsevolodvolodin@yandex.ru (V.V.V.); gskrasnov@mail.ru (G.S.K.); alina.bogdashka@yandex.ru (A.S.B.); nlbolsheva@mail.ru (N.L.B.); fedorowams@yandex.ru (M.S.F.); rhizamoeba@mail.ru (A.V.K.); 2 Institute of Geography, Russian Academy of Sciences, Staromonetny Pereulok, 29/4, 119017 Moscow, Russia; 3 Center for Precision Genome Editing and Genetic Technologies for Biomedicine, Engelhardt Institute of Molecular Biology, Russian Academy of Sciences, Vavilov Str., 32, 119991 Moscow, Russia; 4 Department of Biology, Lomonosov Moscow State University, 119234 Moscow, Russia

**Keywords:** *Populus alba*, rhizosphere, soil metagenomics, microbial diversity, amplicon sequence data, 16S rDNA

## Abstract

The white poplar (*Populus alba*) is a dioecious woody plant with significant potential for the phytoremediation of soils. To realize this potential, it is necessary to utilize growth-promoting microorganisms. One potential source of such beneficial microorganisms is the rhizosphere community of wild-growing trees. However, the structure, dynamics, and metabolism of the rhizosphere community of wild-growing white poplar remain poorly understood. To ascertain seasonal dynamics, species diversity, and metabolic potential, we sequenced 16S rRNA genes in metagenomes derived from 165 soil samples collected in spring and autumn from the root surfaces of 102 trees situated in disparate geographical locations. The three most prevalent phyla across all samples are Proteobacteria, Actinobacteriota, and Acidobacteriota. At the order level, the most prevalent orders are Sphingomonadales and Rhizobiales. Accordingly, the families Sphingomonadaceae and Rhizobiaceae were identified as dominant. The rhizospheric microbiome exhibited substantial inter-seasonal variation. Six families, including Caulobacteraceae, Xanthomonadaceae, Chitinophagaceae, Chthoniobacteraceae, Sphingomonadaceae, and Rhizobiaceae, exhibited alterations (spring-to-autumn) across all geographical locations under study. Members of the Rhizobiaceae family, which includes nitrogen-fixing bacteria, can provide poplar with plant-available forms of nitrogen such as nitrate and ammonium. The rhizosphere microbiome may facilitate the conversion of inorganic sulfur into sulfur-containing amino acids, cysteine and methionine, that are bioavailable to plants. Furthermore, the rhizosphere microbiome is capable of synthesizing amino acids, organic acids (including Krebs cycle acids), and some lipids and sugars. Consequently, the rhizosphere community can stimulate poplar growth by providing it with readily available forms of nitrogen and sulfur, as well as building blocks for the synthesis of proteins, nucleic acids, and other macromolecules. Many of these pathways, including nitrogen fixation, were subjected to seasonal changes.

## 1. Introduction

Phytoremediation is a promising alternative for cleaning up contaminated soils and restoring ecosystems. White poplar (*Populus alba* L.) is a popular woody plant among researchers and has become a model for studying the mechanisms of plant-microbe interactions in woody plants. Poplar has a wide range of practical applications: it is used for timber production, its rapid growth makes it useful in combating climate change, and it is planted on urban and industrial soils for phytoremediation and landscaping due to its ability to transfer and accumulate cadmium and nickel from the environment into its tissues [1].

It is known that microorganisms surrounding plants can modulate stress tolerance and enhance defense responses [2,3], including biological control by preventing the spread of pathogenic bacterial strains [4]. Poplar has a branched root system that provides a large surface area for contact with the soil and habitat. In addition, the roots contain many nutrients such as sugars, amino acids, and carbohydrates, and are thus ideal attachment sites for various microorganisms [5]. Moreover, the microbial community is dynamic and changes depending on the nutrient content of the soil or the presence of metabolites released by plant tissues [6]. Poplar roots contain endophytes (fungi, bacteria, and archaea within the plant tissue) and rhizosphere microorganisms (those on the root surface) that help the host plant to fight pathogens and phytophages, resist abiotic stresses, including heavy metals and organic pollution, and stimulate growth by secreting hormones, etc. [5,7].

In addition, bacteria can also be a bioremediation tool [8]. It is important to consider that one part of the microbial community may be sensitive to metal pollution [9,10] while the other part may be resistant to pollution, so when the soil is contaminated they become dominant, able to mobilize the undigested form of metal into an available form for uptake and accumulation by plants [11]. In this context, strategies to develop and exploit plant-microorganism relationships to enhance plant resistance to pollution [12] and to increase the efficiency of neutralization of heavy metals from soils [13,14] have been considered in recent years.

A major problem in studying such interactions is the lack of sufficient data on how the composition of the plant rhizosphere environment varies with growing region and season. The lack of such data is an obstacle to the development of bacterial-plant co-culture technology, as it is unclear which community members are stably represented regardless of environmental conditions. Thus, characterizing the complex interactions between poplar and its microorganisms is an important step in understanding the general properties of plants, and studying the poplar rhizosphere also helps to elucidate which microorganisms and soil processes influence its phytoremediation capacity. Understanding the changes that occur in this community in response to environmental conditions will help to develop strategies to improve the phytoremediation efficiency and resilience of white poplar to adverse factors.

The aim of the study was to determine the species diversity, seasonal dynamics, and metabolic potential of the rhizosphere community of wild white poplar growing in different regions of European Russia.

## 2. Materials and Methods

### 2.1. Sample Collection

Soil samples were collected for this study from 104 trees growing in natural populations in three regions of the Russian Federation: Volga Federal District (Nizhny Novgorod Region, suburb of Nizhny Novgorod, PFO), Southern Federal District (Volgograd Region, suburb of Uryupinsk, UFO), and North Caucasus Federal District (Stavropol Territory, suburb of Pyatigorsk-Podkumok River valley, SKFO). Female and male individuals were represented among the trees, as well as trees whose sex could not be determined. To assess the variability of rhizosphere bacterial community, soil samples were collected two times a year: in spring (April–May) and in autumn (October–November) in 2022. The general characteristics of the samples are given in Table 1. The sex of trees from which samples were collected and their growing region are indicated. Each line corresponds to one region, for which the number of trees included in the analysis and the number (spring and autumn) of the corresponding samples are indicated. The complete sample data are summarized in Table S1.

Soil samples were collected at a depth of 10 cm from the soil surface. Rhizosphere samples were obtained using a laboratory spoon from the root surface, stored, and transported in 2 mL tubes containing MagNA Pure DNA Tissue Lysis Buffer (F. Hoffmann-La Roche Ltd., Basel, Switzerland). Following collection, samples were stored and transported at −20 °C. The collected soil samples were stored at −75 °C prior to DNA extraction.

The soils of the research regions had the following characteristics: Nizhny Novgorod region—sod-meadow loamy alluvial soil (fluvisol), pH 5–5.5, total carbon 2–2.5%; Uryupinsk region—sod-meadow sandy alluvial soil (fluvisol), pH 4.5–5, total carbon 0.5–1%; Sochi region—sod-meadow loamy alluvial soil (fluvisol), pH 5.5–6, total carbon 2–2.5%; Pyatigorsk region—sod-meadow loamy alluvial soil (fluvisol), pH 8, total carbon 1.5–2%.

### 2.2. DNA Library Preparation and Sequencing

DNA was isolated from a soil sample (average mass about 250–300 mg) according to the standard protocol of the MagnoPrime kit (NextBio, Moscow, Russia). DNA quantification was performed on a Qubit 2.0 fluorometer (Thermo Fisher Scientific, Waltham, MA, USA), and quality control was performed on a NanoDrop ND-1000 spectrophotometer (NanoDrop Technologies Inc., Wilmington, DE, USA). The A260/A280 ratio in DNA samples was 1.8–2.0.

Amplicon libraries were prepared according to the two-step PCR protocol described previously [16]. Briefly, in the first step, target sequences were amplified using primers containing locus-specific sequences for the V3–V4 region of the 16S rRNA gene (16S Amplicon PCR Forward Primer: CCTACGGGGGNGGCWGCWGCAG; 16S Amplicon PCR Reverse Primer: GACTACHVGGGTATCTAATCC) [17,18]. For each sample, amplicons were pooled equimolarly and a second PCR was performed with Nextera XT Index primers consisting of two-index barcodes and sequencing adapters. All PCR products were then pooled equimolarly, and library quality was assessed using an Agilent 2100 Bioanalyzer (Agilent Technologies, Santa Clara, CA, USA) and quantity using a Qubit 4 fluorimeter (Thermo Fisher Scientific, Waltham, MA, USA). Library sequencing was performed on a MiSeq platform (Illumina, USA) using Illumina MiSeq Reagent Kit v3 (150 cycles). An average of 20 thousand reads (from 1.5 to 82 thousand) per sample were obtained.

### 2.3. Data Analysis

Briefly, the sequencing data were processed by DADA2 [19], and the list of microbial taxa was inferred using naïve RDP classifier (DADA2) and Silva 138.1 database [20]. Because of short read length, bootstrap confidence threshold was used at 0.5. Prediction of metabolic potential of bacterial community was based on PICRUSt2 [21] and MicFunPred [22]. Alpha diversity indices were calculated using ‘vegan’ package [23] at the family level. Differential taxon abundance analysis was performed with ALDEx2 [24]. Due to the pronounced heterogeneity of the groups, we mainly relied on the Mann–Whitney U test (either from ALDEx2 or simply normalized abundance values), but Welch’s *t*-test was also considered to detect any potential microbiome changes.

## 3. Results

**Figure 1:**
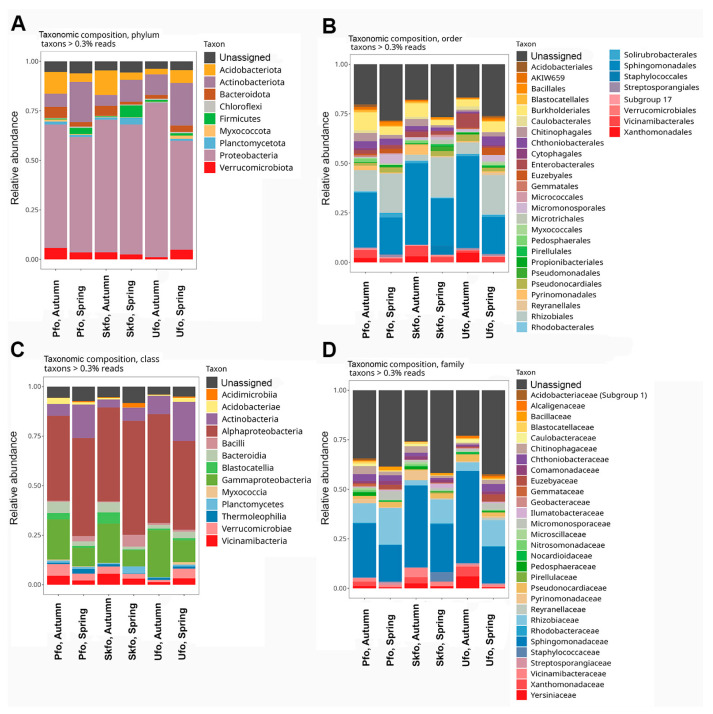
Taxonomic composition of the rhizosphere microbiome in different regions in fall and spring: PFO, UFO, and SKFO. Analyses were performed for taxa of different levels. (**A**) phyla; (**B**) classes; (**C**) orders; (**D**) families. Reads corresponding to unannotated amplicon sequence variants (ASVs) are highlighted in dark gray.

Using 16S rRNA metagenome sequencing data, we first assessed the taxonomic diversity of soil microbiomes at various taxonomic levels (Figure 1, Table S2). Depending on the taxonomic level, the soil samples differed from each other to various degrees. Nevertheless, it was possible to identify dominant taxonomic groups characteristic of most specimens. The top three dominant phyla in all samples are Proteobacteria, Actinobacteriota, and Acidobacteriota. The top three dominant classes are Alphaproteobacteria, Gammaproteobacteria, and Actinobacteria. At the order level, the Sphingomonadales and Rhizobiales are clearly dominant. Accordingly, the dominance of the families Sphingomonadaceae and Rhizobiaceae was found.

It should be noted that the lower the taxonomic level, the higher the content of unassigned ASVs and unidentified taxons. This is expected and is mainly due to the ambiguity of 16S-amplicon sequences or, in some rare cases, the presence of new taxa not present in the reference database.

The data indicate that certain taxa exhibit lower abundance in the spring than in the autumn. For instance, the class Gammaproteobacteria and the Sphingomonadaceae family are less prevalent in the spring than in the autumn. Conversely, other taxa are more abundant in the autumn than in the spring. The class Actinobacteria and the family Rhizobiaceae are examples of this phenomenon.

**Figure 2:**
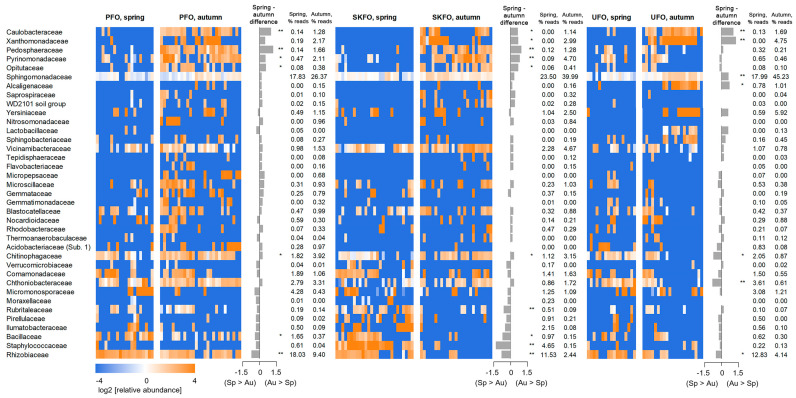
Most abundant bacterial families in poplar rhizospheres collected in three regions of Russia during the spring and autumn seasons. An asterisk (*) indicates taxa with statistically significant (*p* < 0.05 by the Mann−Whitney test, Aldex2) inter-seasonal changes, two asterisks (**) indicate taxa with FDR < 0.05. Heatmaps show log2 of relative taxon abundance (normalized to the average value across all the samples). ‘Spring-autumn difference’ shows difference between autumn and spring, considering intra-group standard deviation (median effect size, Aldex2). Positive values mean that taxon abundance increases in autumn compared to spring, and vice versa. The two columns on the right represent the average percentage of reads annotated as a current taxon for spring and autumn samples.

Evidence suggests that the composition of the rhizosphere community may vary not only due to the physiological characteristics of individual plants, but also due to geographic and climatic differences [25]. Accordingly, we proceed with a comparative analysis of the rhizosphere microbiome composition between different geographical regions and seasons (Figure 2). The data obtained revealed significant differences, both between seasons and between geographic regions.

Despite the existence of discernible inter-seasonal and inter-geographical differences, the variation in microbiome structure types within each group remained considerable. Nevertheless, several discernible cross-regional dissimilarities were identified. In many cases, these differences exhibited a pronounced seasonal dependence. Consequently, numerous taxa exhibited a sufficiently high presence in one location during both spring and autumn, whereas in another location, they were observed predominantly during one season (autumn or spring). For instance, members of the bacterial family Rhizobiaceae were identified at PFO in both seasons, representing 85–100% of samples. However, at SKFO, they were predominantly observed in spring (90% of samples), with a significantly lower prevalence in autumn (15% of samples). Similarly, at UFO, Rhizobiaceae were more prevalent in spring, yet the proportion of Rhizobiaceae-positive specimens remained relatively high in autumn.

Additionally, the “strict” region-specific families were identified. Thus, the presence of Staphylococcaceae was observed exclusively in the SKFO spring samples (90% of spring samples; 4.6% of reads on average), whereas these taxa were almost entirely absent in the other two regions (as well as in SKFO autumn). Caulobacteraceae were predominantly identified in the autumn samples from PFO and UFO, representing 80% of the total samples, while no other groups exhibited the same level of presence. In the spring season, as well as during both seasons in SKFO, the proportion of Caulobacteraceae-positive samples was low (10–30%). Additionally, among the families with notable geographical differences in abundance, Vicinamibacteraceae exhibited a higher abundance in PFO and SKFO, while Pyrinomonadaceae demonstrated a higher content in PFO and SKFO, particularly during the autumn season. In the springtime, as well as during both seasons in SKFO, the proportion of Caulobacteraceae-positive samples was low (10–30%). Additionally, among the families with geographical differences in abundance, Vicinamibacteraceae (higher abundance in PFO and SKFO) and Pyrinomonadaceae (higher content in PFO and SKFO, mainly during the autumn season) stand out. 

The most abundant phyla that exhibit statistically significant changes in content between regions during the autumn season are Acidobacteriota, Verrucomicrobiota, and Bacteroidota (FDR < 0.05). It is noteworthy that in the autumn period, SKFO and PFO exhibited a high content of these phyla in nearly all samples, whereas only 20–30% of UFO samples demonstrated this trend. For the spring period, differences between SKFO and UFO were also identified, but the nature of these differences was markedly distinct. In the case of Acidobacteriota and Bacteroidota, the results were inversely correlated, with higher abundance observed in UFO samples compared to SKFO.

Furthermore, at least six families exhibited statistically significant alterations (at least one of the tests; *p* < 0.05) between spring and autumn in all three regions: Caulobacteraceae, Xanthomonadaceae, Chitinophagaceae, Chthoniobacteraceae, Sphingomonadaceae, and Rhizobiaceae. The latter family of bacteria is of particular interest as it encompasses a range of plant symbionts that enhance plant growth.

**Figure 3:**
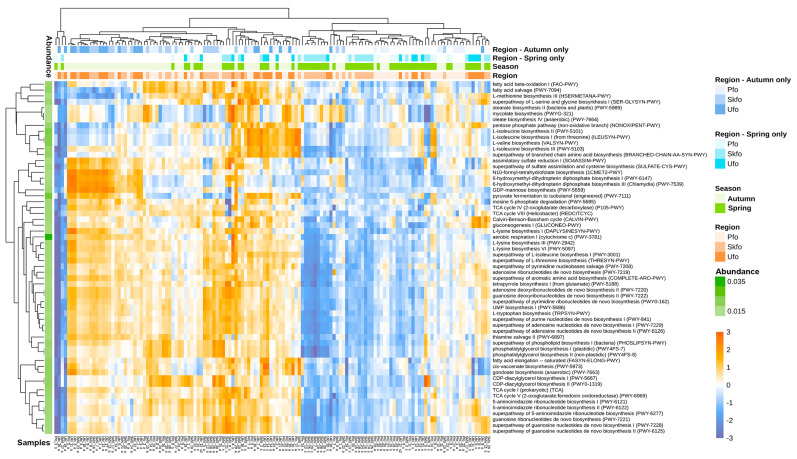
Assessment of the metabolic potential of the rhizosphere microbiome for individual samples using PICRUSt2 (top−60 most abundant MetaCyc pathways). The color scale (blue−white-orange) indicates the log-relative abundance (availability) of a metabolic pathway (i.e., all genes required for a given pathway must be present in the microbial community).

Microbial communities associated with poplar may provide valuable metabolites that could alter the metabolism of trees [26]. Therefore, we estimate the metabolic potential of the poplar-associated rhizosphere microbiomes (Tables S3–S7). The results presented in Figure 3 show that among the 60 most common pathways, there are several that may be beneficial to poplars. These include pathways of sulfate assimilation, which are associated with the synthesis of cysteine and methionine. These pathways may provide poplar with readily available sources of sulfur forms. The most commonly represented pathways are those involved in amino acid and nucleotide biosynthesis. The other abundant pathways are those involved in lipid biosynthesis and the biosynthesis of organic acids that are components of the Krebs cycle (all tricarbonic acids of the cycle).

Regarding the interseasonal and interregional differences in the abundance of metabolic pathways of the rhizosphere microbiome (Figure 3 and Figure S1), it can be seen that in all three regions, metabolic pathways are less abundant in spring than in autumn, which correlates well with the reduced abundance of the rhizosphere microbiome in spring (Figure 1 and Figure 2).

## 4. Discussion

We found that the rhizosphere microbiome of wild white poplar is enriched with chemoorganotrophic and photosynthetic bacteria characteristic of different soils and geographical locations. The families Sphingomonadaceae and Rhizobiaceae were identified as dominant in all geographic locations and seasons. In general, taxa abundance is lower in spring than in autumn at all geographic locations. Metabolic potential of the rhizosphere microbiome showed significant interseasonal fluctuations and were lower in spring than in fall.

Among the taxa identified in the rhizosphere microbiome at the family level, several families are notable for their beneficial effects on poplars (Figure 1). For example, representatives of the Bacillaceae family have been observed to possess antifungal activity [27], stimulate plant growth, denitrify, and degrade organic pollutants [28]. Methylobacteriaceae has been demonstrated to induce systemic resistance to pathogens, in addition to its function of stimulating plant growth [29,30]. Staphylococccaceae plays a role in mitigating biotic stresses [31]. Moraxellaceae is responsible for denitrification and the degradation of organic pollutants [28]. The rhizosphere microbiome of wild poplar is dominated by representatives of the well-known and important family of symbiotic bacteria Rhizobiales, which perform the role of nitrogen fixers [32]. It is established that the family Rhizobiales is more frequently detected when studying bacterial communities of woody plants [33]. In legumes, representatives of this family form nodules; however, in woody plants, although they do not contribute to the formation of nodules, they are also capable of fixing atmospheric nitrogen and converting it into nitrate and ammonium forms available to the plant [34].

The present study demonstrates that there are seasonal variations in the abundance of the rhizosphere community (Figure 1). Prior research has indicated a decline in the diversity of microbiomes associated with trees, including poplar, during the autumn season [35,36,37]. Further investigation reveals that representatives of the Moraxellaceae, Bacillaceae, Propionibacteriaceae, Enterobacteriaceae, Methylobacteriaceae, Staphylococcaceae, and Streptococcaceae families are among the top 10 bacterial taxa exhibiting seasonal declines in diversity [37]. Several potential explanations for this observed phenomenon can be put forth. First, it may be a change in carbon sources for the symbionts. In the autumn, poplars shed their leaves, thereby terminating the flow of organic matter from the crown to the root. Concurrently, the source of organic matter shifts to the fallen leaves, which are subsequently recycled by saprophytes. Second, saprophytes that process forest litter, such as mold fungi, release mycotoxins that inhibit microbial growth [38]. Consequently, the rhizosphere microbes must adapt to the alteration of the carbon source and the competition for food with other organisms during the autumn season. These causes may result in a reduction in the diversity of the rhizospheric microbiome due to the mortality (or transition to a dormant state) of susceptible species and an increase in the abundance of other species that are capable of adapting to the aforementioned conditions. Furthermore, some bacterial families and genera with seasonal dynamics are characteristic of freshwater ecosystems. For instance, the autumnal shift in the prevalence of the family Caulobacteraceae within the class Alphaproteobacteria is a notable example. Given that white poplar is a species that flourishes in river valleys and wet soils, it is plausible that the dynamics of such bacterial communities are linked to spring floods.

The observed changes in rhizosphere composition of representatives of bacterial families for SKFO and PFO are quite symmetrical and similar, especially for those bacteria whose representation increases significantly by autumn (Figure 2). The rhizosphere community of poplars from the UFO behaves quite differently, in which the increase of representatives of individual families (such as Pedosphaeraceae, Opitutaceae, and Pyrinomonadaceae) is not so pronounced. This is an interesting observation as all three regions are geographically distant from each other. We can relate the characteristic behavior of the rhizosphere community of SFO to the periodic waterlogging of the Khoper River, which occurs in this white poplar growing region. It is known that periodic waterlogging negatively affects the development of beneficial members of the microbiome, allowing the development of phytopathogenic ones [39]. We can confirm that, by autumn, the decrease in the diversity of bacteria of the Rhizobiaceae family, potentially favorable for poplar physiology, is more pronounced in UFO. At the same time, the diversity of species of the Xanthomonadaceae family (which includes the known plant bacteriosis pathogen genus Xanthomonas) increases more pronouncedly. This observation undoubtedly requires more careful monitoring, since poplars prefer to grow in the valleys of water bodies. Moreover, we cannot exclude that anomalous temperatures observed at the time and places of sample collection [40] could also contribute to the inter-regional differences in the composition of the rhizosphere communities

The results of the evaluation of the metabolic potential of the rhizosphere (Figure 3) are in good agreement with existing literature, which suggests that in the absence of a soil microbiome (when poplar is grown on sterilized soils), poplar needs increased amounts of amino acids, organic acids (including Krebs cycle acids), and some lipids and sugars, so there is increased biosynthesis of these substances in poplar roots [26]. Our data also suggest that the rhizosphere community capable of incorporating sulfur sources in metabolism is higher than in the autumn. Sulfur is a constituent of amino acids, and also contributes to plant resistance to salt stress [41] and enhances the action of phytohormones [42]. In general, plants can assimilate sulfur from soil by their roots [43]. However, it remains unclear whether bacteria contribute to this process or, conversely, compete for this macronutrient. What is evident, however, is that the process of sulfur assimilation or mineralization by the bacterial community should be more active in the spring than in the autumn.

The relatively low metabolic potential of the rhizosphere community in spring (Figure S1) may be associated with the delayed onset of the climatic spring, which results in a delayed onset of community development. It should be noted that this does not imply a reduction in community diversity. Based on these observations, we propose that the rhizosphere community is highly sensitive to the environmental conditions in which the trees are cultivated.

## 5. Conclusions

In the present study, plant-growth promoting taxa were observed in rhizosphere communities associated with the wild-growing white poplar. These taxa exhibited geographical and interseasonal differences in abundance, composition, and metabolic potential. Our observations suggest that these differences are environmentally driven rather than related to the phenological phase of the plants. Further longitudinal investigation is required to test this hypothesis, with weather and other environmental factors to be monitored.

## Figures and Tables

**Table 1:** General characteristics of the rhizosphere samples analyzed.

Federal District	Trees Included in the Analysis *			Rhizosphere Samples		Total Included in the Analysis	
	Male	Female	Unspecified	Spring Period	Autumn Period	Trees	Rhizosphere Samples
PFO	12	18	0	22	30	30	52
UFO	7	8	6 **	16	21	21	38
SKFO	7	17	6 **	27	25	30	52

* Sex was determined by inspection of the tree in the presence of generative organs or by PCR for the *ARR17* sex gene with primers as described in [15]. ** Visual inspection of one tree from the region revealed both male generative organs and female generative structures simultaneously.

## Data Availability

The data presented in this study are openly available in NCBI SRA repository and have an identification number: PRJNA1072085 (SRP487950). Direct URL to data: https://www.ncbi.nlm.nih.gov/bioproject/PRJNA1072085 (accessed on 24 September 2024).

## Supplementary Materials

The following supporting information can be downloaded at: https://www.mdpi.com/article/10.3390/biotech13040052/s1, Table S1: Characteristics of rhizosphere samples analyzed; Table S2: 16S—taxonomic data, normalized read counts; Table S3: Picrust2 enzyme (EC) abundance normalized 0–100%; Table S4: MicFuncPred enzyme (EC) abundance normalized 0–100%; Table S5: Picrust2 KEGG KO gene abundance nor-malized 0–100%; Table S6: MicFunPred MetaCyc reactions abundance normalized 0–100%; Table S7: Picrust2 MetaCyc pathways abundance normalized 0–100%; Figure S1: Assessment of seasonal differences in the metabolic potential of the rhizosphere microbiome using PICRUSt2 (top-90 most differentiated MetaCyc pathways).
