# Supplementary material for: Composition, Seasonal Dynamics and Metabolic Potential of the Rhizosphere Microbiome Associated with Wild White Poplar
Source: BioTech (Basel). 2024 Dec 1;13(4):52. doi: 10.3390/biotech13040052 (PMC11674042; doi:10.3390/biotech13040052)
Supplement: Supplementary file 1 [file biotech-13-00052-s001.zip › Supplementary Figure S1.pdf]

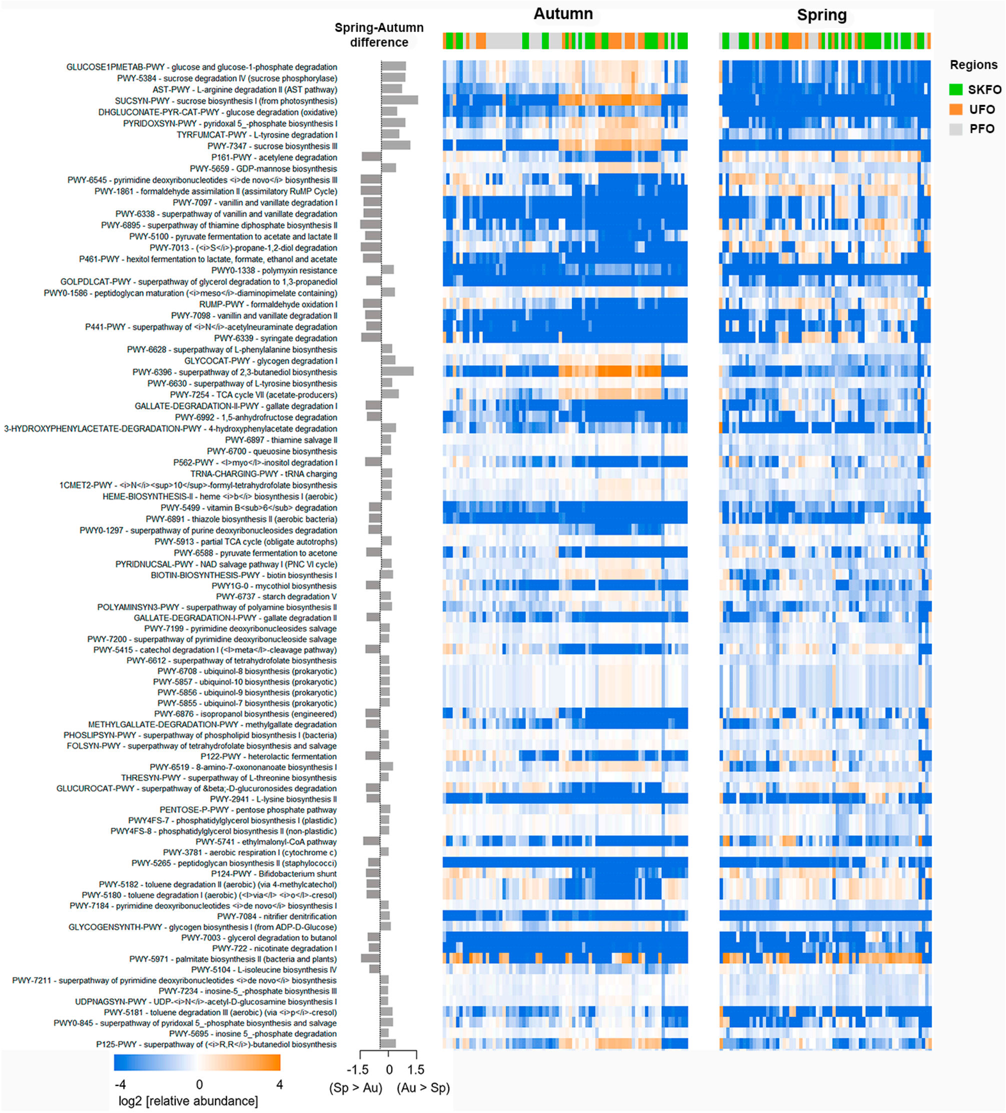

Figure S1: Assessment of seasonal differences in the metabolic potential of the rhizosphere microbiome using PICRUSt2 (top-90 most differentiated MetaCyc pathways).
