# Supplementary material for: Composition, Seasonal Dynamics and Metabolic Potential of the Rhizosphere Microbiome Associated with Wild White Poplar
Source: BioTech (Basel). 2024 Dec 1;13(4):52. doi: 10.3390/biotech13040052 (PMC11674042; doi:10.3390/biotech13040052)
Supplement: Supplementary file 1 [file biotech-13-00052-s001.zip › Supplementary Table S1.pdf]

Supplementary Table S1. Characteristics of rhizosphere samples analyzed, ontogenetic stage and sex of trees from which samples were collected and their geographical data. Each row corresponds to a single tree. For each tree, a spring rhizosphere sample ("+" in the "Spring Sample" column), an autumn sample ("+" in the "Autumn Sample" column), or both samples were collected.

Sample name	Geographic region and habitat soil types	Sampling location	Spring sample	Autumn sample	Tree sex*
cfo-14	City of Moscow, Urbic Technosol	55.826599, 37.493121	+	-	Generative, Female
cfo-16	City of Moscow, Urbic Technosol	55.793023, 37.556559	+	-	Generative, Female
cfo-27	City of Moscow, Urbic Technosol	55.836725, 37.552391	+	-	Generative, Female
cfo-30	City of Moscow, Urbic Technosol	55.835864, 37.567393	+	-	Generative, Generative, Male
cfo-33	City of Moscow, Urbic Technosol	55.836212, 37.566489	+	-	Generative, Male
cfo-39	City of Moscow, Urbic Technosol	55.836578, 37.554478	+	-	Generative, Female
cfo-41	City of Moscow, Urbic Technosol	55.667621, 37.59773	+	-	Generative, Female
cfo-44	City of Moscow, Urbic Technosol	55.665872, 37.58988	+	-	Generative, Male
cfo-45	City of Moscow, Urbic Technosol	55.665865, 37.589644	+	-	Generative, Male
cfo-51	City of Moscow, Urbic Technosol	55.674999 37.566241	+	-	Generative, Female
cfo-54	City of Moscow, Urbic Technosol	55.674999 37.566241	+	-	Generative, Male
cfo-55	City of Moscow, Urbic Technosol	55.674999 37.566241	+	-	Generative, Female
cfo-76	City of Obninsk, Urbic Technosol	55.092946, 36.618006	+	-	Generative, Male
cfo-79	City of Obninsk, Urbic Technosol	55.093126, 36.617936	+	-	Generative, Female
cfo-80	City of Obninsk, Urbic Technosol	55.093165, 36.617957	+	-	Generative, Female
cfo-86	City of Obninsk, Urbic Technosol	55.093294, 36.617994	+	-	Generative, Female
cfo-89	City of Obninsk, Urbic Technosol	55.093348, 36.618076	+	-	Generative, Female
cfo-93	City of Obninsk, Urbic Technosol	55.093393, 36.618043	+	-	Generative, Female
cfo-94	City of Obninsk, Urbic Technosol	55.093426, 36.618069	+	-	Generative, Female
cfo-100	City of Obninsk, Urbic Technosol	55.09408, 36.61837	+	-	Generative, Female

cfo-101	City of Obninsk, Urbic Technosol	55.094135, 36.618377	+	-	Generative, Female
cfo-103	City of Obninsk, Urbic Technosol	55.094214, 36.618388	+	-	Generative, Female
cfo-106	City of Obninsk, Urbic Technosol	55.093859, 36.619888	+	-	Generative, Female
Pfo_1	Suburb of Nizhny Novgorod city, floodplain of the Volga River, Fluvisol	56.352374, 44.059665	+	+	Generative, Female
Pfo_2	Suburb of Nizhny Novgorod city, floodplain of the Volga River, Fluvisol	56.352357, 44.059899	-	+	Generative, Female
Pfo_3	Suburb of Nizhny Novgorod city, floodplain of the Volga River, Fluvisol	56.352267, 44.059949	+	+	Generative, Female
Pfo_4	Suburb of Nizhny Novgorod city, floodplain of the Volga River, Fluvisol	56.352254, 44.060078	+	+	Generative, Female
Pfo_5	Suburb of Nizhny Novgorod city, floodplain of the Volga River, Fluvisol	56.352335, 44.060471	-	+	Generative, Female
Pfo_6	Suburb of Nizhny Novgorod city, floodplain of the Volga River, Fluvisol	56.350187, 44.057717	+	+	Generative, Female
Pfo_7	Suburb of Nizhny Novgorod city, floodplain of the Volga River, Fluvisol	56.350513, 44.055672	+	+	Generative, Female
Pfo_8	Suburb of Nizhny Novgorod city, floodplain of the Volga River, Fluvisol	56.350367, 44.056690	+	+	Generative, Female
Pfo_9	Suburb of Nizhny Novgorod city, floodplain of the Volga River, Fluvisol	56.349087, 44.059552	+	+	Generative, Male

Pfo_10	Suburb of Nizhny Novgorod city, floodplain of the Volga River, Fluvisol	56.34895, 44.059657	+	+	Generative, Female
Pfo_11	Suburb of Nizhny Novgorod city, floodplain of the Volga River, Fluvisol	56.349175, 44.059504	+	+	Generative, Female
Pfo_12	Suburb of Nizhny Novgorod city, floodplain of the Volga River, Fluvisol	56.348399, 44.059859	+	+	Generative, Female
Pfo_14	Suburb of Nizhny Novgorod city, floodplain of the Volga River, Fluvisol	56.347917, 44.059613	+	+	Generative, Female
Pfo_15	Suburb of Nizhny Novgorod city, floodplain of the Volga River, Fluvisol	56.347924, 44.057064	+	+	Generative, Male
Pfo_16	Suburb of Nizhny Novgorod city, floodplain of the Volga River, Fluvisol	56.34864, 44.05762	+	+	Generative, Male
Pfo_17	Suburb of Nizhny Novgorod city, floodplain of the Volga River, Fluvisol	56.348846, 44.057411	-	+	Generative, Male
Pfo_18	Suburb of Nizhny Novgorod city, floodplain of the Volga River, Fluvisol	56.34865, 44.057088	+	+	Generative, Male
Pfo_19	Suburb of Nizhny Novgorod city, floodplain of the Volga River, Fluvisol	56.348595, 44.056844	+	+	Generative, Male
Pfo_20	Suburb of Nizhny Novgorod city, floodplain of the Volga River, Fluvisol	56.349736, 44.053389	+	+	Generative, Male
Pfo_21	Suburb of Nizhny Novgorod city,	56.347224, 44.060078	-	+	Generative, Female

	floodplain of the Volga River, Fluvisol				
Pfo_22	Suburb of Nizhny Novgorod city, floodplain of the Volga River, Fluvisol	56.347372, 44.061504	+	+	Generative, Male
Pfo_23	Suburb of Nizhny Novgorod city, floodplain of the Volga River, Fluvisol	56.346653, 44.061317	+	+	Generative, Male
Pfo_24	Suburb of Nizhny Novgorod city, floodplain of the Volga River, Fluvisol	56.346479, 44.061316	-	+	Generative, Female
Pfo_26	Suburb of Nizhny Novgorod city, floodplain of the Volga River, Fluvisol	56.346533, 44.06196	-	+	Generative, Female
Pfo_27	Suburb of Nizhny Novgorod city, floodplain of the Volga River, Fluvisol	56.348623, 44.062902	-	+	Generative, Female
Pfo_28	Suburb of Nizhny Novgorod city, floodplain of the Volga River, Fluvisol	56.349537, 44.062455	-	+	Generative, Male
Pfo_29	Suburb of Nizhny Novgorod city, floodplain of the Volga River, Fluvisol	56.349836, 44.061572	+	+	Generative, Male
Pfo_30	Suburb of Nizhny Novgorod city, floodplain of the Volga River, Fluvisol	56.350171, 44.059993	+	+	Generative, Male
Skfo_1	Podkumok River Valley, Fluvisol	44.027603 43.067865	+	-	Generative, Female
Skfo_2	Podkumok River Valley, Fluvisol	44.027049 43.06349	+	-	Generative, Female
Skfo_3	Podkumok River Valley, Fluvisol	44.027473 43.06353	+	-	Generative, Male

Skfo_4	Podkumok River Valley, Fluvisol	44.027473 43.06353	+	+	Generative, intersex**
Skfo_5	Podkumok River Valley, Fluvisol	44.04294 42.932274	-	+	Generative, Male
Skfo_6	Podkumok River Valley, Fluvisol	44.04294 42.932274	+	-	Generative, Female
Skfo_7	Podkumok River Valley, Fluvisol	44.043669 42.933025	+	+	Generative, Male
Skfo_8	Podkumok River Valley, Fluvisol	44.043904 42.933252	+	+	Generative, Female
Skfo_9	Podkumok River Valley, Fluvisol	44.045263 42.935257	+	+	Generative, Female
Skfo_10	Podkumok River Valley, Fluvisol	44.045182 42.938949	+	+	Generative, Female
Skfo_11	Podkumok River Valley, Fluvisol	44.04521 42.939581	+	+	Generative, Male
Skfo_12	Podkumok River Valley, Fluvisol	44.045228 42.939812	+	+	Generative, Male
Skfo_13	Podkumok River Valley, Fluvisol	44.044609 42.942218	+	-	Generative, Female
Skfo_14	Podkumok River Valley, Fluvisol	44.03778 42.943411	+	+	Virginile, Not Defined
Skfo_15	Podkumok River Valley, Fluvisol	44.045557 42.944202	+	+	Virginile, Not Defined
Skfo_16	Podkumok River Valley, Fluvisol	44.044412 42.946224	+	+	Virginile, Not Defined
Skfo_17	Podkumok River Valley, Fluvisol	44.042521 42.963468	+	+	Generative, Male
Skfo_18	Podkumok River Valley, Fluvisol	43.957302 42.768589	+	+	Generative, Female
Skfo_19	Podkumok River Valley, Fluvisol	43.955697 42.766329	+	+	Generative, Female
Skfo_20	Podkumok River Valley, Fluvisol	43.954759 42.765917	+	+	Generative, Female

Skfo_21	Podkumok River Valley, Fluvisol	43.954701 42.765658	+	+	Generative, Female
Skfo_22	Podkumok River Valley, Fluvisol	43.954502 42.765205	-	+	Virginile, Not Defined
Skfo_23	Podkumok River Valley, Fluvisol	43.954949 42.766178	-	+	Generative, Female
Skfo_24	Podkumok River Valley, Fluvisol	43.959573 42.773464	+	+	Generative, Female
Skfo_25	Podkumok River Valley, Fluvisol	43.959558 42.774582	+	+	Virginile, Not Defined
Skfo_26	Podkumok River Valley, Fluvisol	43.963678 42.781233	+	+	Generative, Female
Skfo_27	Podkumok River Valley, Fluvisol	43.967392 42.785425	+	+	Generative, Female
Skfo_28	Podkumok River Valley, Fluvisol	43.971874 42.789184	+	+	Generative, Female
Skfo_29	Podkumok River Valley, Fluvisol	43.972696 42.789123	+	+	Generative, Female
Skfo_30	Podkumok River Valley, Fluvisol	43.973228 42.788866	+	+	Generative, Male
Ufo_1	Suburb of Uryupinsk city, Khopyor river valley, Fluvisol	50.789744 41.988373	+	+	Generative, Female
Ufo_2	Suburb of Uryupinsk city, Khopyor river valley, Fluvisol	50.787686 41.985166	+	+	Virginile, Not Defined
Ufo_3	Suburb of Uryupinsk city, Khopyor river valley, Fluvisol	50.787714 41.984997	-	+	Generative, Female
Ufo_4	Suburb of Uryupinsk city, Khopyor river valley, Fluvisol	50.790325 41.978117	+	+	Virginile,Not Defined
Ufo_5	Suburb of Uryupinsk city, Khopyor river valley, Fluvisol	50.79067 41.977279	+	+	Generative, Male

Ufo_6	Suburb of Uryupinsk city, Khopyor river valley, Fluvisol	50.791846 41.978294	+	+	Virginile, Not Defined
Ufo_7	Suburb of Uryupinsk city, Khopyor river valley, Fluvisol	50.793013 41.979879	-	+	Generative, Female
Ufo_8	Suburb of Uryupinsk city, Khopyor river valley, Fluvisol	50.793133 41.979933	-	+	Generative, Male
Ufo_9	Suburb of Uryupinsk city, Khopyor river valley, Fluvisol	50.791472 41.984088	-	+	Generative, Male
Ufo_10	Suburb of Uryupinsk city, Khopyor river valley, Fluvisol	50.813251 41.992686	+	+	Generative, Female
Ufo_11	Suburb of Uryupinsk city, Khopyor river valley, Fluvisol	50.811365 41.987989	+	+	Generative, Female
Ufo_12	Suburb of Uryupinsk city, Khopyor river valley, Fluvisol	50.811208 41.988316	+ (2 samples)	+	Virginile, Not Defined
Ufo_13	Suburb of Uryupinsk city, Khopyor river valley, Fluvisol	50.810635 41.980038	+	+	Generative, Male
Ufo_14	Suburb of Uryupinsk city, Khopyor river valley, Fluvisol	50.810218 41.979661	+	+	Generative, Female
Ufo_15	Suburb of Uryupinsk city, Khopyor river valley, Fluvisol	50.810313 41.975727	+	+	Generative, Female
Ufo_16	Suburb of Uryupinsk city, Khopyor river valley, Fluvisol	50.784777 41.986269	+	+	Generative, Male
Ufo_17	Suburb of Uryupinsk city, Khopyor river valley, Fluvisol	50.784834 41.986221	+	+	Generative, Male
Ufo_18	Suburb of Uryupinsk city, Khopyor river valley, Fluvisol	50.782144 41.984908	-	+	Virginile, Not Defined

Ufo_19	Suburb of Uryupinsk city, Khopyor river valley, Fluvisol	50.785039 41.985022	+	+	Generative, intersex**
Ufo_20	Suburb of Uryupinsk city, Khopyor river valley, Fluvisol	50.786221 41.986017	+	+	Generative, Male
Ufo_21	Suburb of Uryupinsk city, Khopyor river valley, Fluvisol	50.787698 41.985552	+	+	Generative, Female

* Sex was determined by inspection of the tree when generative organs were present or by PCR to the ARR17 sex-specific gene.

** Visual inspection of the tree revealed both male generative organs and female generative structures at the same time.
